# Reconstructing the 2003/2004 H3N2 influenza epidemic in Switzerland with a spatially explicit, individual-based model

**DOI:** 10.1186/1471-2334-11-115

**Published:** 2011-05-09

**Authors:** Timo Smieszek, Michael Balmer, Jan Hattendorf, Kay W Axhausen, Jakob Zinsstag, Roland W Scholz

**Affiliations:** 1 Institute for Environmental Decisions, Natural and Social Science Interface, ETH Zurich, Universitaetsstrasse 22, 8092 Zurich, Switzerland; 2 Institute for Transport Planning and Systems, ETH Zurich, Wolfgang-Pauli-Strasse 15, 8093 Zurich, Switzerland; 3 Department of Public Health and Epidemiology, Swiss Tropical and Public Health Institute, Socinstrasse 57, 4002 Basel, Switzerland; 4 University of Basel, P.O. Box, 4003 Basel, Switzerland

## Abstract

**Background:**

Simulation models of influenza spread play an important role for pandemic preparedness. However, as the world has not faced a severe pandemic for decades, except the rather mild H1N1 one in 2009, pandemic influenza models are inherently hypothetical and validation is, thus, difficult. We aim at reconstructing a recent seasonal influenza epidemic that occurred in Switzerland and deem this to be a promising validation strategy for models of influenza spread.

**Methods:**

We present a spatially explicit, individual-based simulation model of influenza spread. The simulation model bases upon (i) simulated human travel data, (ii) data on human contact patterns and (iii) empirical knowledge on the epidemiology of influenza. For model validation we compare the simulation outcomes with empirical knowledge regarding (i) the shape of the epidemic curve, overall infection rate and reproduction number, (ii) age-dependent infection rates and time of infection, (iii) spatial patterns.

**Results:**

The simulation model is capable of reproducing the shape of the 2003/2004 H3N2 epidemic curve of Switzerland and generates an overall infection rate (14.9 percent) and reproduction numbers (between 1.2 and 1.3), which are realistic for seasonal influenza epidemics. Age and spatial patterns observed in empirical data are also reflected by the model: Highest infection rates are in children between 5 and 14 and the disease spreads along the main transport axes from west to east.

**Conclusions:**

We show that finding evidence for the validity of simulation models of influenza spread by challenging them with seasonal influenza outbreak data is possible and promising. Simulation models for pandemic spread gain more credibility if they are able to reproduce seasonal influenza outbreaks. For more robust modelling of seasonal influenza, serological data complementing sentinel information would be beneficial.

## Background

Mathematical models and computer simulations of influenza spread have become increasingly important for pandemic preparedness within the last few years and have influenced the decisions of public health authorities [1,2]. A non-systematic search in the common publication databases identified plenty of studies modelling the spread of (mostly pandemic) influenza outbreaks [3-13]. However, models of pandemic spread are in most cases hypothetical because they focus on future pandemics [e.g. [6,7,10-13]] and, thus, are not validated with empirical data. In contrast, some models of historical case examples explicitly address the model validation issue [e.g. [5]], but they are of limited value for understanding nowadays pandemic threat as society has changed vastly. As there is no alternative to prospective, hypothetical modelling for addressing scientifically potential future problems, new strategies for model validation are needed.

By validation we understand confirming that a certain model provides a good reproduction of the real-world behaviour we are trying to simulate [14]. In agreement with the prevailing view in philosophy of science, we see model validation as a rather non-algorithmic, but argumentative process [15,16]: Achieving a good statistical fit between a simulated and an empirical epidemic curve does not automatically mean that the model is valid. Instead, it has to be agreed upon which aspects of reality shall be reproduced and what reproduction exactly means.

The more real world data sets can be reproduced with a certain model or the more known characteristics can be reproduced with a model, the more reason we have to believe that the model is valid. Inherently, models forecasting future events cannot be checked against empirical data, but applying such a model successfully to past events provides some certainty that the model is valid *per se *and can be used for the comparative assessment of different scenarios including interventions.

This validation strategy has successfully been applied in various fields, such as climate research [17] or disease spread, where Carpenter and Sattenspiel [5] reconstructed meticulously the characteristics of the 1918 influenza outbreak in an indigenous Canadian community by means of an individual-based model. The problem when influenza pandemic models are challenged with data about past pandemics is that the last pandemics date back so far that they are only of limited value as mobility patterns and contact structures changed vastly.

An alternative approach is to challenge simulation models with data from seasonal influenza outbreaks. Seasonal influenza outbreaks feature some particularities, which increase the system complexity and make them quite often resistant against attempts to reproduce them successfully in simulation models: Most pandemic models assume that there is no pre-existing host immunity due to the novelty of a pandemic strain [e.g. [8-10]], but in the case of seasonal influenza this cannot be ignored. Further, seasonal influenza epidemics are often characterized by several co-existent strains, which have to be treated as distinct diseases but which can interact in complex manner at the same time. Nevertheless, challenging models with seasonal influenza data is a promising validation strategy, because for seasonal influenza we have topical data.

In this paper, we present and describe a spatially explicit, individual-based model of influenza spread in Switzerland. We chose an individual-based approach because this allows us to include spatial and social heterogeneities - important factors for understanding patterns of disease - easily. The model makes use of disaggregated human travel data of whole Switzerland generated by the open source transport simulation software MATSim [18]. We further select data from the 2003/2004 H3N2 influenza epidemic in Switzerland and delineate why it is a good example for reconstruction and model validation. Our aim is to show that the simulation outcome is consistent with measured data and empirically based knowledge about the following aspects of seasonal influenza:

1.) Epidemic curve, overall infection rate and reproduction number

2.) Age-dependent infection rates and time of infection

3.) Spatial patterns of influenza spread.

## Methods

In this section, we first substantiate why we chose to model the 2003/2004 H3N2 influenza epidemic for model validation. We then describe the available data on this epidemic in Switzerland. Finally, we present all social and biological processes and assumptions which constitute our simulation model of influenza spread (see also Additional File 1).

### Choosing the influenza season to be reconstructed

Seasonal influenza epidemics are very complex in their dynamics and their effective drivers. On top, there are enormous uncertainties regarding the involved transmission processes, pre-existing host immunity, the proportions of asymptomatic, mild, moderate and severe courses of infection as well as the "true" infection rates. For a successful reconstruction of a preceding epidemic, a careful selection of the epidemic is highly important. The epidemic should have certain properties that reduce the complexity to a manageable level and that make it well suited for model validation. We present three of such properties subsequently.

1. *Only one dominant influenza strain in the period of interest*. Quite often we see two or more strains circulating simultaneously in a population. Having more than one strain at a time leads to an increase in system complexity that cannot be tackled in a simulation model: Two different strains must be modelled as two distinct diseases, but usually existing sentinel data only monitors on the basis of ILI symptoms and does not differentiate by strain. Therefore, there is no data on spatial patterns and age dependency of different strains. In Switzerland from the eleven influenza seasons between 1995 and 2006 just three fulfilled the criterion of having a dominant strain which is responsible for almost all analyzed cases: 1997/1998; 1999/2000 and 2003/2004 (cf. Table 1).

**Table 1:** Frequency of different influenza strains in analyzed Swiss samples

Season	A H1N1		A H1N2		A H3N2		B		Total
	Cases	proportion	cases	proportion	cases	proportion	cases	proportion	
1995/96	146	51%		0%	109	38%	30	11%	285
1996/97	2	1%		0%	234	68%	109	32%	345
1997/98	5	2%		0%	321	98%		0%	326
1998/99		0%		0%	83	37%	143	63%	226
1999/00		0%		0%	115	100%		0%	115
2000/01	110	89%		0%	1	1%	13	10%	124
2001/02		0%	1	0%	103	44%	130	56%	234
2002/03	1	1%	5	3%	125	68%	52	28%	183
2003/04	1	0%		0%	225	99%	2	1%	228
2004/05	35	12%		0%	225	75%	41	14%	301
2005/06	9	4%		0%	13	6%	183	89%	205

The data has kindly been provided by the Swiss Federal Office of Public Health.

**Figure 1:**
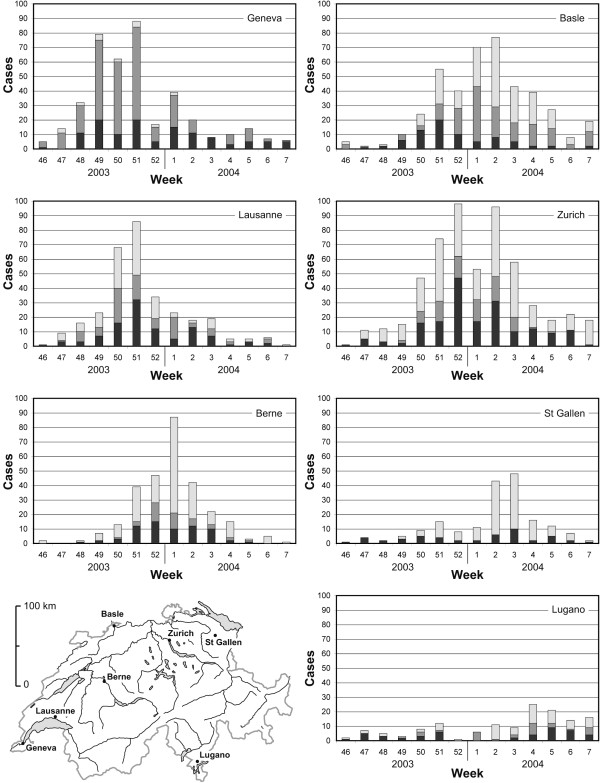
**Reported cases of seven Swiss cities and their hinterland**. The figure shows the reported cases of (i) seven Swiss cities (black bars); (ii) municipalities, whose centre is in a range of 7.5 km from the centre of the respective city (dark grey bars); (iii) municipalities with a centre in the range of 15 km (light grey bars)

**Figure 2:**
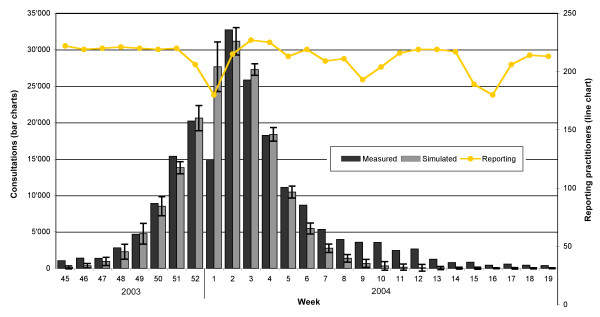
**Epidemic curves and reporting practitioners**. The dark grey bars show the extrapolated reported cases coming from the Swiss sentinel system. The light grey bars show the average simulated number of cases. The whiskers represent the standard deviation. The orange line stands for the number of reporting practitioners during the course of time.

2. *Clear empirical spatial patterns can serve as one indicator amongst others for the validity of a simulation model*. If there are observable spatial differences in the course of an influenza epidemic, these differences can be tried to be reproduced. If a simulation model reproduces observed spatial-temporal patterns (amongst other characteristics), then this is a further cue that the structural decisions and model assumptions underlying the simulation are appropriate. On the European continent, in several seasons a clear west-east-direction of influenza spread was observable [19]. Such a west-east trend can also be observed in several years within Switzerland. However, of all the years with a single dominant strain, this west-east trend is most pronounced in the 2003/2004 season.

3. *Inflows of infected persons from outside the system boundaries should not affect the internal spread dynamics in a relevant manner*. Every simulation model has system boundaries - typically national borders. In case of Switzerland there are between 167000 (2003) and 213000 (2008) cross-border commuters [20], who travel daily from Germany, France, Italy and Austria to Switzerland and who are potentially an important source of imported influenza infections. The condition that the dynamics should be primarily internal is met by the 2003/2004 H3N2 epidemic. The epidemic took off in the westernmost part of Switzerland around Geneva during the 47th and 48th calendar weeks of 2003 (cf. Figure 1) - preceded by the westernmost countries in Europe (England, Scotland, Portugal and Spain peaked already during the 45th and 46th calendar week) -, where it probably was introduced from France [19,21]. Within Switzerland the epidemic moved towards the east within the next few weeks with a nationwide peak in the second week of 2004 (cf. Figures 1 and 2). Spatial dynamics seen within Switzerland might be mainly governed by internal processes because the other two neighbouring countries of Switzerland with a long common border, Germany and Italy, experienced only an extremely mild and late influenza season. Germany had much less excess physician consultations than in the four preceding seasons and Baden-Württemberg (the German federal state neighbouring Switzerland) peaked in the 9th week of 2004 when the epidemic in Switzerland was almost over [21]; Italy as a whole peaked in the 6th week [19].

### Swiss influenza sentinel data

Approximately 3% (some 200; cf. Figure 2, orange line) of the general practitioners participate voluntarily in the Swiss sentinel system. They report the number of ILI diagnoses and the total number of consultations in their surgery on a weekly basis [22]. The nationwide incidence of influenza cases leading to a consultation is calculated by the Swiss Office of Public Health based on this sentinel data and the nationwide number of consultations known from the health insurance companies. The extrapolated number of consultations due to influenza for the 2003/2004 H3N2 epidemic in Switzerland is shown in Figure 2 (dark grey bars).

The Swiss influenza sentinel data further allow coarse spatial analyses as every reported case is linked to a concrete practitioner and as for every practitioner it is known to which municipality she or he belongs.

### Transmission pathways of influenza and contact definition

Known transmission pathways for human influenza viruses include direct physical contact, indirect physical contact, direct transmission via large droplets or indirectly by aerosols. No consensus has been achieved about the relative importance of these pathways under natural conditions. Some deem aerosols an important path [23-25] while others emphasize that close interaction is usually needed for transmission [26,27]. For example, a past influenza outbreak on an airplane was best explained with aerosol transmission [28], whereas a recent H1N1 outbreak appeared to be caused solely by close, direct conversation [29]. It is also possible that the relative importance of the pathways depends on climatic conditions and virus strain specific characteristics.

The decision on which pathway of transmission a model should be based, has far-reaching implications for the modelling strategy: aerosol transmission would be best represented by dynamic affiliation networks (individuals contaminate places; cf. [30]) and the transmission risk depends vastly on ventilation conditions and less on social interaction [31]. Transmission by large droplets and physical contact are best described by dynamic one-mode networks (individuals infect individuals directly, cf. [30]); the related risk of infection can be estimated based on intensity and duration of the respective contact [32].

Here, we subscribe to the assumption that transmission via large droplets and close contact are the dominant pathways of transmission and thus direct and close interaction between individuals is a precondition for infection in our model. Information on contact duration and intensity is not included in the current version of the model, instead all contacts weigh equally.

### Spatio-social contact structure

#### Identifying sets of potential contact partners

Understanding and reproducing the spatial patterns of human movements is the common interest of spatially explicit epidemic models and transport simulation models. Hence, it is reasonable to link transport modelling and epidemiological modelling in a synergistic way, as has previously been done e.g. for smallpox spread in the Portland region [33,34] or for respiratory diseases [35]. For identifying individuals that possibly have contact with each other, we utilize a validated [36,37] open source toolbox called MATSim [18], which was developed for implementing large-scale agent-based transport simulations. Details on MATSim are given elsewhere [18,38,39].

**Figure 3:**
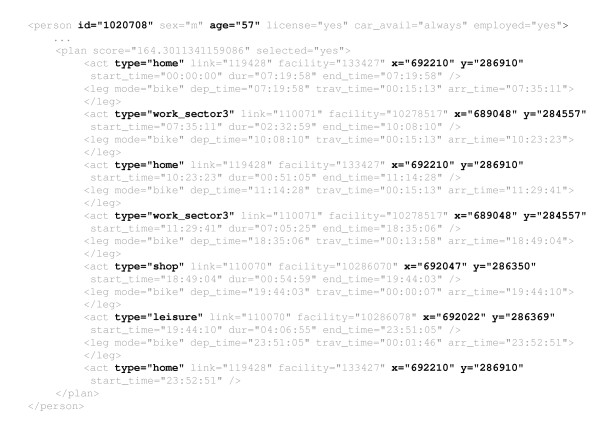
**MATSim output - Sequence of activities**. This figure shows an excerpt of the XML data structure generated by MATSim. The highlighted information is used in the here presented influenza model: **id **refers to an existing unique identifier in the Swiss census; **age **gives the age in years of the respective agent; every activity has a **type **- we distinguish home, work, education, shop and leisure; **x **and **y **are coordinates and refer to the Swiss military grid.

The wish or the need to perform certain activities is the main driver for human movement [39]. Individuals choose activities on different locations. That generates traffic and potentially infectious contacts [38]. Sequences of activities ("schedules") are generated and equilibrated in MATSim based on information about the characteristics of every individual of the (synthetic) population, i.e. their needs and preferences, and about the social and built environment, e.g. opening hours, (cf. Table 2; the use of the data sources presented in Table 2 was approved by the responsible authorities) [38,39]. The MATSim simulation process has three steps: (i) scenario creation (establishing a synthetic population [39], the transport network [40] and the set of locations with their corresponding capacities and open times), (ii) initial demand modelling (creating an initial schedule for every individual based on given data [41]) and (iii) demand equilibration (allowing individuals iteratively to re-decide on their schedules for achieving more realistic results) [39]. Compared to state-of-practice transport modelling processes [42] MATSim comes up with an integrated assignment and demand equilibration completely based on time-dynamic schedules for every member of the synthetic population. These individual schedules are saved in an XML-file (an excerpt of such a file is shown in Figure 3) that can be used for further analysis or as input data for other simulations - like in the epidemic model presented in this paper.

**Table 2:** Sources of information used for the Swiss MATSim scenario

Source	Description and information used
Volkszählung 2000	The Swiss census is conducted once a decade and includes the entire legal resident population (~7.2 million in 2000). It links inhabitants to concrete buildings and households and provides information about demographical characteristics, place(s) of residence, place of work, etc.
Mikrozensus Verkehr 2005	The "Mikrozensus Verkehr" is a travel behaviour survey carried out by the Swiss Federal Statistical Office every five years. In 2005 a sample of 33390 inhabitants participated and answered questions about their mobility behaviour. In particular, they filled in a travel and activity diary.
Betriebszählung 2000	The enterprise census ("Betriebszählung") records data on all public or private enterprises in Switzerland. There are ~398000 workplaces in Switzerland and for all of them the exact location, the sector they belong to and the number of employees is known.
Nationales Netzmodell	The national transportation planning network consists of ~24000 nodes and 60000 links of the Swiss transportation grid.

Based on this information, we can identify individuals, who share the same location and thus having a chance to infect each other. In the current version of our model we map all individuals on a geographic grid with a side length of 500 by 500 meters. In principle, the set of potential contact partners for each individual consists of those people, who perform the same activity within the same grid cell. However, for some activity categories, we further narrowed down these sets as follows (see also Table 3):

**Table 3:** Groups of potential contact partners

Activity categories	Group definition
Home	All individuals living in one household.
Work	1) Grid cell group: all individuals working within a grid cell^1^.
	2) Work group: all individuals working within one grid cell^1 ^are randomly assigned to work groups. The target group size distribution is a normal distribution with a mean of 10 (SD = 2).
Education	1) Grid cell group: all individuals performing an educational activity within a grid cell^1^.
	2) Class A: all individuals aged 18 or younger within one grid cell^1 ^are assigned to classes. The target class size is 20 students and classes are stratified by age.
	3) Class B: all individuals older than 18 within one grid cell^1 ^are randomly assigned to classes. The target class size is 20 students. There is no age stratification.
Shop	All individuals shopping within a grid cell^1^.
Leisure	All individuals performing leisure activities within a grid cell^1 ^(not at home).

^1^The physical space is divided into grid cells of 500 by 500 meters.

##### Home contacts

We assume that every individual has daily contact with every other individual who lives in the same household. Information on the exact household compositions is taken from the Swiss census (cf. Table 2). Contacts at home beyond the regular cohabitants are not included in the simulation model.

##### Work contacts

All individuals who work within a certain grid cell are allocated to work groups. The targeted work group sizes are assumed to follow a normal distribution with a mean of ten work group members and a standard deviation of two (values rounded to the nearest integer value; see also Additional File 1). Every individual can only belong to one work group per grid cell. If there are not enough individuals in a grid cell to form a workgroup of the targeted size, they form a workgroup of the maximally possible size. All individuals within the same work group as well as the whole set of working individuals within the same grid cell are eligible as contact partners according to the selection rules explained subsequently.

##### Educational contacts

All individuals who perform any kind of education activity within a certain grid cell are allocated to classes. Based on the data published in the education statistics of the canton Zurich [43], we assume that 20 students form a school class (see Additional File 1). Up to an age of 18, only students of the same age can form a class. Educational activities of adults include everything from university to evening classes. For these individuals, we also form classes of 20 - but these classes are not stratified by age. All individuals within the class as well as the whole set of individuals within the same grid cell performing education activities are eligible as contact partners according to the selection rules explained next.

##### Contacts during shopping or leisure time

For both categories shopping and leisure, we assume no further structure: All individuals, who perform either shopping or leisure activities within a certain spatial cell, are eligible as contact partners for another individual with the respective activity within this cell.

#### Realizing contacts

Relying on the data from the Swiss MATSim scenario, we were able to identify sets of potential contact partners for every individual. However, people do not have contact every day with all persons they could possible have contact with: Students do not have contact with the same classmates every day and employees have meetings with different co-workers each day. Thus, only a subset of the potential contact partners has to be chosen every simulation time step as realized contacts.

To do so, we rely on age-dependent distributions of potentially contagious contacts measured in ten European countries [44]. We calculate the mean number of household contacts (all other household members) for each age group and subtract it from the empirical means [44], which results in the remaining average number of contacts to be achieved during the daily non-home activities. By calculating how many distinct activities are on average performed by the members of a certain age group, we can figure out how many contacts have to be made in average per activity in order to reach the contact number targets. We assume that the contact distribution per activity and age group follows a negative binomial distribution [44]. Details are given in Table 4.

**Table 4:** Contact data

Age group	**Empirical mean **[44]^**1**^	Mean household contacts in CH^2^	Mean activities per person^3^	Mean contacts per activity [SD]^4^	Median contacts per activity [IQR]^4^
0-4	10.21	2.95	1.29	5.62 [4.0]	5 [3-8]
5-9	14.81	3.23	1.52	7.6 [5.2]	7 [4-10]
10-14	18.22	3.23	1.54	9.75 [6.4]	9 [5-13]
15-19	17.58	2.98	2.29	6.38 [4.5]	6 [3-9]
20-29	13.57	2.12	2.06	5.56 [4.0]	5 [3-8]
30-39	14.14	2.40	1.91	6.14 [4.3]	5 [3-8]
40-49	13.83	2.52	1.90	5.95 [4.2]	5 [3-8]
50-59	12.30	1.63	1.87	5.70 [4.1]	5 [3-8]
60-69	9.21	1.32	1.72	4.58 [3.4]	4 [2-6]
70+	6.89	1.39	1.82	3.02 [2.5]	3 [1-4]

^1^Average number of contact partners per day as measured by Mossong et al.

^2^Average number of contact partners within the individuals' household. Calculations are based on the Swiss census (Volkszählung 2000) and we assume that all individuals living in one household interact in a potentially contagious manner on a daily base.

^3^Average number of distinct activities performed by one individual per day.

^4^We assume that the number of contacts per activity follows a negative binomial distribution. The two columns describe the distributions that were determined to achieve a plausible overall contact distribution (see the "Realizing contacts" section).

Based on the so-defined probability distributions, we allocate a target number of contact partners to each distinct combination of a person performing a certain activity at a certain location. The allocation is a random process taking place newly at every simulation time step and completely independent from former allocations. To note, individuals, who perform many activities meet on average more other people than individuals with only few activities. With the Swiss MATSim scenario we achieve in total an average number of contacts per day of 12.4 (SD = 8.9; mode = 7), which is plausible [44].

The target numbers of contact partners allocated as described are like stubs which have to be connected in order to become contacts. We connect stubs on demand, i.e. we look only for contact partners for those individuals, who are infectious. If all of a certain individual's stubs are connected to other individuals' stubs, this individual is no longer available as a contact partner.

In principle, contact partners are chosen from the set of other individuals performing the same activity within the same grid cell. In addition, for two out of four non-home activities fixed subgroups have been defined: Employees are allocated to work groups and students are allocated to classes. We adopt the assumption of Ferguson et al. and assume that with a probability of 0.75 the counterpart for an unconnected stub comes from the work group or the class, respectively, whereas the others are chosen from the total set on the grid cell level (cf. p. 8 of the supplementary material of [8]). As we know that more contacts are made with people of the approximately same age than with people, who are considerably younger or older [44,45], we introduced a slight age-dependent bias for the partner selection out of the total set: The relative probability weight of being picked is *P *= exp(*c *Δ_*age*_), where Δ_*age *_is the age difference (in years) of two potential contact partners and *c *is a shape parameter modulating how sensitive the selection process is for age differences. We assume *c *= -0.02.

Approximately 45 percent of all contacts are made within the defined subgroups (household, classes and work groups) - which appears us to be realistic [46]. If there are no stubs left within the group or if all individuals within the group have already established contacts with the individual to be connected, the contact partner will be automatically chosen from the total set of potential partners. If there are not enough partners left in the total set, we allow connecting to individuals from adjacent grid cells (von Neumann neighbourhood). This exemption occurs in less than 2 per thousand of all individuals to be connected.

### Pre-existing immunity, acquired immunity and vaccination

Modelling seasonal influenza, in contrast to pandemic influenza, must account for patterns of pre-existing partial or full immunity. For Switzerland no such measures like the pre-episode hemagglutination-inhibiting (HI) antibody titres (cf. table five of [47]) are available. Therefore, we assume here that individuals older than 20 years to have a halved transmission risk [48], i.e. we multiply the transmission probability for every contact between an infector and a susceptible individual older than 20 years by 0.5.

A part of the Swiss population gets vaccinated against the dominant seasonal influenza strains every year. Several sources report vaccination efficacy values between 70 and 90 percent [48-50]. In this paper, we assume vaccination to reduce the per contact transmission probability by 80 percent for all age groups. We do not distinguish further components of vaccine efficacy (e.g. reduction in infectivity) as the available data is not accurate enough [51].We had no data about the age distribution of vaccinated people in Switzerland for 2003/2004, but as the overall vaccination rates (and vaccination strategies) are similar in the neighbouring countries [52] we took the age distribution of Germany [53] and adjusted it proportionally to achieve approximately 1.31 million doses (see also Additional File 1) - a realistic number for whole Switzerland [54].

After recovery, we assume individuals to be fully immune for the rest of the simulation run, i.e. they cannot be re-infected.

### Severity of infection and shedding

Only a minor proportion of all seasonal influenza infections leads to such severe illness that a practitioner's advice - or even hospitalization - is needed. The yearly infection rates reported in the literature range between 5 and 30 percent (most of them between 10 and 20 percent) [49,55-58], but only approximately 2-3 percent of the Swiss population seek out a general practitioner due to an ILI during the influenza season every year.

Influenza occurs with varying severity and can be even completely asymptomatic [59]. The exact proportion of asymptomatic infection and of the different levels of severity is unknown. Modelling studies used proportions of asymptomatic infection between 30 and 50 percent [6,8,9,60-62]. Wright et al. reported an asymptomatic infection rate of 37 percent in children [63]. Carrat et al. re-analysed volunteer challenge studies and found that the proportion of infected individuals showing any kind of symptoms was 66.9 percent (CI: 58.3, 74.5) - however, only 34.9 percent (CI: 26.7, 44.2) reported fever [64]. Fox et al. [47] showed that the proportions of different levels of severity depend on age and pre-episode hemagglutination-inhibiting (HI) antibody titre.

In addition, the relative infectiousness of asymptomatic, mild, moderate and severe cases is not known. The infectiousness can be represented as a function of the viral shedding [32], quite often simplified as a linear relation [8,64]. There is only little known about the shedding behavior of asymptomatic cases (and, thus, about their infectiousness) [59,64]. However, in two studies a positive correlation was found between the quantity of virus per positive specimen and the reported severity of illness [65,66]. For the case of the 1918/19 pandemic influenza in Geneva, Chowell et al. estimated the relative infectiousness of asymptomatic cases to be negligibly small [67]. Ferguson et al. assume severe cases to be twice as infectious as mild cases based on household data [8,9]. Eichner et al. assume asymptomatic cases to be half as infectious as moderate or severe cases [68]. The empirical work of Couch et al. showed that the mean quantity of virus detected in specimens of nasal wash was only approx. half in case of asymptomatic infection compared to symptomatic infection (cf. figure one of [66]).

For this paper we decided to distinguish two groups of illness severity: We assume that 50 percent of the infections end up in moderate to severe illness (subsequently referred to as symptomatic cases) and that 50 percent of the infections are asymptomatic or very mild cases (referred to as asymptomatic cases). We further assume that the latter group is half as infectious as the first group. Although in reality there is an interaction between age, pre-existing immunity and illness severity, we simplify here and assume illness severity to be a random outcome completely independent of the other two factors.

### Incubation period and infectiousness over time

Anderson and May specify the incubation period of influenza as between one and three days [69]. Lange and Vogel deem the onset of symptoms to be 24 hours after inoculation (cf. figure three.two in [49]). Most empirical studies report the onset of symptoms to be within the first one or two days after inoculation [8,64]. In our simulation model the step width is one day, i.e. we can implement the course of a disease with such a precision. We assume here - simplifying reality - that the latency period equals the incubation period and that virus shedding always starts the day after inoculation.

Various numbers for the infectious period can be found in literature: Anderson and May assume the infectious period to be two to three days [69]. Wearing et al. use for their model of an influenza outbreak in an English boarding school infectious periods of 2.1 to 2.2 days (depending on the make of the model) [70]. Eichner et al. implement a "fully contagious period" of 4.1 days for asymptomatic and moderately sick adults and of 7.0 days for all other groups [68]. However, most simulation models assume the infectiousness to be constant during the infectious period. We describe the infectiousness over time by a function estimated by Ferguson et al. [9] based on shedding data: They assume the infectiousness over time to follow a lognormal function *κ*(*T,δ,γ*) with *δ*[log(*d*)]= -0.72 and *γ*[log(*d*)]= -1.8 (for details cf. to pp. 10 and table SI1 of the supplementary material of [9]; see also Additional File 1). We truncated this function after seven days for both the symptomatic and the asymptomatic group.

The total infectiousness (over the seven days of infection) can be varied by multiplying the values for every single day by a constant factor. The previously presented model parameters are listed in Table 5.

**Table 5:** Model parameters for the 2003/2004 influenza epidemic in Switzerland

Relative susceptibility	
Individuals aged 20 or younger	100%
Individuals older than 20	50%
**Vaccination**	
Vaccination efficacy	80%
Doses	1.31 million

**Severity of infection (frequency)**	
Severe and moderate cases	50%
Mild and asymptomatic cases	50%

**Course of infection**	
Incubation period	1 day
Infectious period	7 days

**Relative and absolute infectiousness**	
Severe and moderate cases	100%
Mild and asymptomatic cases	50%
Relative infectiousness over time	lognormal decay with *δ*[log(*d*)]= -0.72 and *γ*[log(*d*)]= -1.8
Absolute per-contact infection probability (averaged over infectious period and assuming 100% infectiousness and 100% susceptibility)	1.1693 10^-2^

### Initial seed

Defining the initial seed is a particularly critical modeling decision and complicated task. Regarding the seasonality of influenza, it is not entirely clear to what degree influenza viruses outlive the summer between two influenza seasons and whether they are reintroduced from other world regions [49]. Usually, seasonal influenza is also prevalent in several regions within Europe at the same time - and therefore multiple ports of entry are possible. Furthermore, the geospatial information coming from sentinel systems is worst at the beginning of an epidemic: In contrast to the epidemic phase during which the positive predictive value of practitioners' diagnoses is around four-fifth [71-73], it is around one-third in absence of a known epidemic in various studies and different age groups [49,74,75]. At the same time, random variation is particularly critical for the further spread during the early phase of an outbreak [76].

We decided to take the sentinel report of the 49th week as starting point for the initialization. For each of the 293 cases reported in this week, an agent with the same age and residential municipality was randomly identified in the synthetic population and symptomatically infected. The actual status in the course of infectiousness was attributed randomly using a uniform probability distribution.

The chosen approach combines various requirements: (i) The 49th week is the first sentinel week with clear signs of an outbreak in the westernmost parts of Switzerland while the rest of Switzerland is still pre-epidemic. Such a "headstart" of the Geneva region is necessary to be able to reproduce the spatial patterns observed. At the same time early cases in the rest of Switzerland are realistically captured by this initialization. (ii) Although the age distribution of the cases reported within the sentinel system is biased (the help seeking behaviour varies across age groups), we can assume that these cases reflect the real age distribution better than e.g. random choice. (iii) 293 initial cases are negligible compared to the total population size and, thus, still a reasonable starting point for model initialization.

### Model calibration

What remains to be calibrated are the overall infectiousness of the disease and the proportion of symptomatic cases which is diagnosed with influenza by a practitioner. We assume the latter proportion to be constant over the entire epidemic season. We further assume perfect diagnosis accuracy, i.e. 100% sensitivity and specificity, and that the extrapolated sentinel data can directly be compared with the respective proportion of the symptomatic cases coming from the simulation runs. We further compare only the weeks 47 to 52 (2003) and 2 to 15 (2004). The first week of 2004 is excluded from the analysis and calibration procedure as we do not know to what degree the observed drop in cases reflects a real decline and to what degree this is a simple measurement error (cf. to the discussion below). In order to compare the weekly sentinel data with the daily simulation data, the latter had to be converted into weekly data, too. We did this with floating week boundaries, i.e. the definition which simulation days should be the beginning of a report week was flexible and chosen in a way to achieve maximum fit with the measured data. The peak of the epidemic was assumed to be in the second week of 2004.

We started our calibration procedure with an arbitrarily chosen mean (i.e. averaged over the seven days an individual stays infectious) per-contact infection probability for symptomatic cases of 1.129.10^-2^. We compared the fit (least squares) of this parameter setting with three higher and three lower values for the mean per-contact infection probability. The increment was 5.65 10^-4 ^(i.e. 5 percent of the start value). The two parameter values that fitted best were the start value and the next higher value (which fitted also far better than the start value). We then analyzed the parameter range between the two best values starting from the higher value and approaching the lower with an increment of 1.6 10^-4^. The best accordance with the empirical epidemic curve was achieved for a mean per-contact infection probability of 1.1693 10^-2^. The proportion of symptomatic cases that has to be diagnosed by a practitioner to reproduce the extrapolated empirical curve best (least squares) is approximately one-third.

## Results and discussion

### Epidemic curve, overall infection rate and reproduction number

#### Epidemic curve

The extrapolated epidemic curve of the 2003/2004 H3N2 epidemic and the corresponding average values and standard deviations of 30 simulation runs are shown in Figure 2. The weeks of the year 2003 and the weeks two to five of 2004 are reproduced quite well by the model. The model overshoots the extrapolated value of the first week of 2004 (began on 29 December 2003) a lot and it undershoots the extrapolated sentinel data of the weeks six to twelve systematically.

There are several explanations for the difference in the first week of 2004 including:

(i) This week is part of the Swiss Christmas vacations and children do not go to school. Children are seen as a major pacemaker for the spread of seasonal influenza [77] and school is one of the most important places to make contact with other children [78,79]. A study conducted by Cauchemez et al. showed a relation between school vacation and a decrease in infection rates in France [80]; Heymann et al. were able to show similar results for the 1999/2000 influenza epidemic in Israel when teachers went on strike during the influenza season [81]. However, another study conducted by Rodriguez et al. failed to show such an association in a survey about absenteeism in King County (Washington) public schools [82]. Hence, there might be a real drop in cases during the Swiss Christmas vacations due to school closure, but such an effect is neither proven nor quantified.

(ii) It is not only plausible to expect fewer infections during school vacations, but also to expect fewer consultations: Many patients with influenza-like symptoms normally do not seek out the practitioner primarily because they want treatment, but for a sick note for school or the employer. As many offices and all schools were closed in this first week of 2004, also many sick people did not need such a medical certificate. Consequently, most likely a smaller proportion of the symptomatic cases sought out a practitioner than usual.

It is further not clear, what causality is behind the undershooting during the tapering phase of the epidemic curve. Plausible explanations are:

(i) The epidemic peaked quite late in the German and Italian neighbourhood of Switzerland. A possible explanation for the comparatively slow decay in the Swiss sentinel data might be that constantly new sources of infection were introduced from the neighbourhood and prevented the disease from dying out earlier. The spatial distribution of the reported cases at the end of the 2003/2004 epidemic appears to support this interpretation as the focal points of infections seems to be at the German and Italian borders. However, such transborder processes are not reflected in our model.

(ii) We have seen that the diagnostic accuracy is good during the peak phase, but comparatively poor when influenza is not abundant. Hence, it is likely that sentinel extrapolations overestimate the number of new cases during the early and the late phase of an epidemic. Particularly at the end of an outbreak after a phase when influenza diagnoses were quite common, practitioners might be prone to diagnose influenza too inattentively.

(iii) It might be possible that sick people are more likely to seek a practitioner's help later in an epidemic when there is widespread recognition that an epidemic is occurring.

(iv) We assume the infectiousness of the infective agent to be constant over time. However, influenza is a quickly mutating virus [83] and we have evidence that the risk of infection is partly modulated e.g. by climatic conditions [84,85]. If - for whatever reason - there was a rise in infectiousness of the 2003/2004 H3N2 strain, this could also explain why the extrapolated measurements decay more slowly than the simulated values.

#### Overall infection rate

The simulated overall infection rate is 14.9 percent for one influenza season. There is no serological data for the 2003/2004 H3N2 epidemic in Switzerland we could compare this simulated value to. However, various sources state that between 5 and 20 percent [57] or between 10 and 20 percent [49] of the population in a Western context become sick of influenza every year, which is the range of our results.

#### Reproduction number

**Figure 4:**
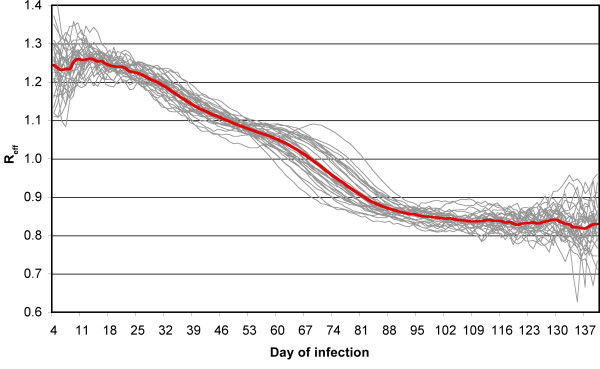
**Reproduction number versus time of infection**. The grey lines show the development of the reproduction number (defined as the average number of secondary cases) of each individual simulation run. The red line is the average of all runs. The abscissa represents the time-point (in days) when an infector started shedding.

In case of seasonal influenza it makes more sense to refer to the effective reproduction number than to the basic reproduction number, because the infectious agent never hits a fully susceptible population and, thus, the basic reproduction number is only of theoretical value. We show the time-development of the reproduction number of our simulation runs in Figure 4. When the disease hits the unaffected population during the early phase of the epidemic, reproduction numbers range between 1.2 and 1.3. This is in accordance with the results reported in studies on influenza spread: Chowell et al. analyzed influenza seasons in the USA, France and Australia from 1972 to 1997 and found for all three countries mean reproduction numbers of 1.3 (CI 1.2-1.4) [57]. Nishiura et al. report maximum likelihood estimates for the reproduction number of the 2009 H1N1 epidemic in Japan between 1.21 and 1.35 [76].

### Age-dependent infection rates and time of infection

#### Infection rates

**Figure 5:**
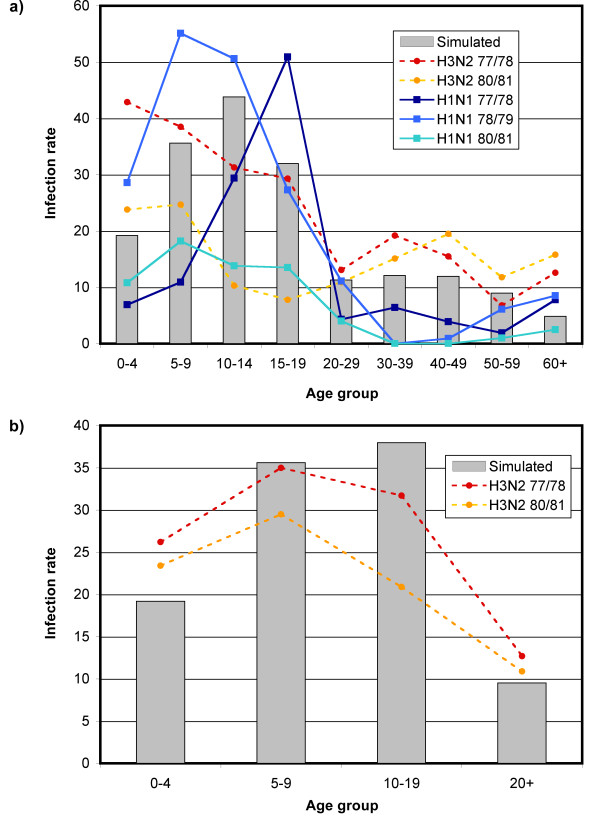
**Age-dependent infection rates**. Subfigure a: The lines show the serological age-dependent infection rates measured for five influenza seasons in Tecumseh, Michigan [86]; the bars show the corresponding average infection rates of our simulation model. Subfigure b: The lines show the serological age-dependent infection rates measures for two influenza seasons in Seattle, Washington [87]; the bars show the corresponding simulated infection rates.

Figure 5 shows the simulated mean age-dependent infection rates. Children (all persons younger than 20) have clearly the highest simulated infection rates with 33.0 percent, whereas adults show only attack rates of 9.5 percent. These simulated results are in accordance with general empirical data on seasonal influenza outbreaks: Several sources state that children are considerably more affected than adults [49,55]. Nicholson et al. estimate that approximately 20 percent of children and 5 percent of adults worldwide develop symptomatic influenza every year [50]. Sauerbrei et al. see the annual infection rates of children in a range between 20 and 30 percent [56].

Figures 5a and 5b further show serologic age-dependent infection rates measured for five influenza seasons in Tecumseh, Michigan, [86] and Seattle, Washington [87]. Although there is a pronounced variability in infection rates, clear patterns are visible in the serologic data, which are also reproduced by the model in a qualitative sense: (i) In most cases, the infection rates of children are twice to three times as high than those of adults. (ii) Very young children and adolescent, almost adult individuals show frequently lower infection rates than children between five and 14 years of age.

The patterns observed in the serologic data as well as in the simulation results can be explained by the following mechanisms: (i) Practically every child over twelve years of age has been challenged with influenza during their lifetime [56]. Every exposure to influenza confers specific immunity against the respective strain and partial immunity against kindred strains. We have implemented this mechanism by assuming that adulthood is 50 percent protective. (ii) Differences in the mean number of contact partners can also explain the observed differences partly. People with many contacts have more chances of infecting others or of becoming infected than people with few contacts. Particularly, the differences within the age group of children can be partly attributed to differences in the number of contact partners (cf. Table 4 and Figures 5a and 5b). (iii) The actual arrangement of contacts also determines infection rates. It is known, for instance, that children tend to have most of their contacts with other children of the same age. If a certain age group has in average more contacts than another, such age assortativeness means that individuals with many contacts preferentially meet also other individuals with many contacts - which results in a further elevated risk of infection.

#### Time of infection

If we relate the average age of infected individuals to their time of infection, we see a clear increase in average age in both sentinel and simulated data. We calculated linear regression models with point in time as independent variable and average age of reported cases as dependent variable.

For the sentinel data, we observe an increase in the average age of reported cases of approx. 0.32 years every day, if we look only at the swelling phase of the epidemic between weeks 49 in 2003 and two in 2004. After the peak in week two has been passed, the average age of the reported cases decreases again. However, for the whole epidemic phase (from week 48 in 2003 to week five in 2004), there is still an increase in the average age of 0.10 years each day. In case of the simulated data, we calculate a median slope of 0.081 years (IQR 0.076- 0.092 of age) per day for the simulation time range between the days 30 and 100.

These increases in the average age of the cases can be explained by the hypothesized pacemaker function of children. Theoretical modelling studies showed that highly connected persons get infected earlier and more often and play a more important role as infectors than rather isolated persons [88,89]. We know from literature [44] and have implemented in our model that children have in average more contact partners per day than adults. The increase in average age by time we see analogously in the sentinel and simulated data is most likely caused by this mechanism: First, the susceptible and highly connected children of a region get infected. Then, the partly-immune and less connected adults follow.

### Spatial spread of the influenza epidemic

The spatial dynamics of the 2003/2004 H3N2 epidemic are visible in Figure 1, which shows the epidemic curves of seven Swiss cities and their hinterland based on the sentinel data. It is clearly observable, that the epidemic broke out in the Geneva region, which is the westernmost part of Switzerland. It moved from there over Lausanne and Berne towards the north-eastern parts of Switzerland with the cities Zurich and St Gallen. At last, it reached the canton of Ticino in the south-east of Switzerland, here represented by the city of Lugano.

**Figure 6:**
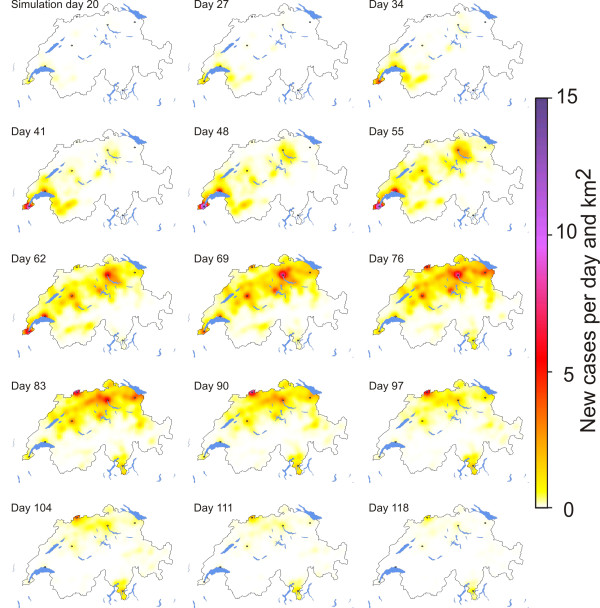
**Simulated spatial outbreak patterns**. This figure shows the prevalence of influenza in the course of simulation time for one simulation run.

Table 6 provides the median duration from the peak in one city to the peak in another city as well as the inter-quartile range as resulted in the 30 simulation runs. The median durations of the 30 simulation runs are consistent with the time lags that can be observed in the measured data. Most of the simulation runs also showed a clear movement from the west to the east with Ticino having the latest of all peaks. One exemplary run is shown in Figure 6 and movies of six selected runs are given in the online Additional Files 2, 3, 4, 5, 6 and 7.

**Table 6:** Median duration from peak to peak and inter-quartile range

	Geneva	Lausanne	Berne	Basle	Zurich	Lugano
St Gallen	4.0 [3.1-4.3]	2.1 [1.3-2.7]	0.4 [-0.4-1.5]	-0.5 [-1.3-0.1]	0.0 [-0.6-0.6]	-2.0 [-3.5--0.9]
Lugano	5.8 [4.8-7.1]	4.5 [3.6-5.6]	2.9 [1.7-3.9]	1.8 [0.2-3.0]	2.4 [1.1-3.0]	
Zurich	3.9 [3.0-4.6]	2.2 [1.2-3.0]	0.5 [-0.1-1.4]	-0.7 [-1.3-0.3]		
Basle	4.6 [3.0-5.4]	2.7 [1.6-3.7]	1.1 [0.1-2.1]			
Berne	3.1 [2.4-4.0]	1.7 [0.8-2.4]				
Lausanne	1.4 [1.1-2.0]					

The epidemic curves were smoothed with a moving average of five days for identifying stable peak days.

These observations regarding the spatial patterns of influenza spread can be interpreted in manifold ways. First, it must be stated that the spatial spread of influenza given certain initial conditions is a stochastic process. Nishiura et al. [76] showed clearly that during the early phase of an outbreak parameters like the reproduction rate can be highly fluctuating - and in a spatially explicit model we observe these random fluctuations at every place where the disease is newly introduced: Before a certain point of no return has been passed *locally*, it is a random process whether the local outbreak dies off again or can be sustained.

It is also a matter of chance whether there is e.g. an infector travelling very early from Geneva to Lugano and causing an outbreak there or whether Lugano is reached rather late. However, we claim that there are typical time lags between the peaks in different regions of Switzerland: There are hubs - like Zurich - which are origin, stopover or final destination for many activities. These hubs are highly connected with the rest of the country by large traffic flows and are, thus, likely to become affected by an epidemic quite early. On the other hand, there are remote regions - like the lower Engadin in the south-western part of the Alps - which have good chances to be spared for quite a long time. The canton of Ticino with the city of Lugano is geographically isolated from the rest of Switzerland by the Alps and strongly oriented towards Italy, which might explain why it was affected last by the 2003/2004 epidemic.

Consequently, we believe that analyzing spatial patterns and cautiously comparing simulated patterns with observed patterns is meaningful, although random variation in both reality and simulation can lead to atypical, i.e. improbable, outcomes.

### Limitations of the model and the study design

#### Limitations of the overall study design

We chose a particular influenza season in order to reconstruct it and justified this case selection. We took the stance that every single aspect of an influenza outbreak which can be reproduced is a cue for the validity of a modelling approach. However, reproducing one particular outbreak should be just a starting point for a validation strategy. Further suitable data sets - such as sentinel data of other influenza seasons or mortality data - should be identified for challenging the proposed influenza model for gaining more certainty that the presented model captures influenza dynamics reasonably well.

Another potential limitation is the number of simulation runs calculated for each parameter setting. However, we calculated the standard errors of the means for critical simulation outcomes (e.g. the overall and the age-dependent infection rates as well as the simulation time until the peak is reached) and all of them were very low. Hence, we conclude that the chosen number of simulation runs is sufficient.

#### Limitations of the empirical data

The reconstruction of a seasonal influenza outbreak in Switzerland rests upon general knowledge about seasonal influenza reported in the literature and upon sentinel information about Switzerland and the surrounding countries.

On the one hand, seasonal influenza is extremely variable in its actual characteristics (cf. e.g. the enormous variability in the age-dependent attack rates shown in Figure 5a), hence, findings reported in older studies can only serve as heuristics and not as precise parameter estimates. Further, one has to note that important factors such as social contact structure vary in different cultural contexts (cf. differences between ten European countries reported in [44]) and, thus, the generalizability of findings to a different social context is limited. Therefore, country-specific data would be needed to adapt the model to other settings.

On the other hand, sentinel data can give only a biased image of reality and have to be interpreted with caution: As mentioned, there is a tendency to overestimate the number of new cases in the early and late phase of an outbreak when true cases are not abundant. Sentinel systems can only record correctly diagnosed, symptomatic cases which seek out a practitioner. Accordingly, all asymptomatic cases and all sick individuals that do not see a practitioner and all false diagnoses remain undetected. Unfortunately, asymptomatic infection is age-dependent and the consultation rates differ also most likely between age groups and between the different parts of Switzerland. Finally, it is not clear whether and - if so - to what degree vacations lead to reporting biases.

For future studies, it would be optimal to have seasonal epidemics for which both sentinel data and serologic information about the overall and the age-dependent infection rate is available. Relying solely on sentinel data is accompanied by a considerable uncertainty regarding the infection rate estimate and age-dependent infection rates as one can only reproduce general knowledge instead of outbreak-specific data.

#### Limitations of the model structure

There are several limitations in the model itself. For instance, it is known that traffic and activity patterns differ vastly between weekdays and weekends [90] - but we assumed weekday patterns throughout the simulation.

We further have included neither voluntary home confinement nor changed patterns due to vacations or other events in our model. We think the former is an acceptable assumption given that we do not know real-world rates of home confinement and given that the severity of symptoms seems to lag the shedding rate slightly [49] and, thus, infectors are likely to expose the community to influenza during their most infectious phase. The latter might be part of the explanation for the difference between the model outcome and the extrapolated sentinel data in the first week of 2004.

Though this could in principle be implemented in our model, we currently also do not distinguish the probability of becoming a symptomatic or asymptomatic case depending on the immunity status of the respective host. We acknowledge that, for instance, the effects of vaccination are much more complex than reducing the probability of becoming infected by a constant factor irrespective of age and pre-existing immunity status. While differentiated vaccination efficacy parameters would be essential in studies on the effect of different vaccination strategies, the impact of our simplification is acceptably low in our concrete setting. We tested how sensitive our results react on changes of the vaccination efficacy and we found that a 25% drop in the assumed efficacy results only in an 8.7% increase in the overall infection rate, i.e. from 14.9% to 16.2%. No considerable changes in the temporal dynamics were observable. These findings can be explained with the low vaccination rates in Switzerland, particularly with the very low rates in young individuals.

Finally, we assume all contacts between an infector and a susceptible person to lead to infection with equal probability irrespective of the duration and the intensity of the contact, which is also known to be a simplification of reality [32]. In our simulation model, the average number of secondary cases per infector is approximately linearly related to the number of contact partners. We believe that this relation is sub-linear in reality.

## Conclusions

This paper had two purposes: (i) A spatially explicit, individual-based model for influenza spread should be introduced and (ii) validated with empirical data from the 2003/2004 H3N2 influenza epidemic. We succeeded in compiling a simulation model, which is capable of reproducing main characteristics of the 2003/2004 H3N2 epidemic in Switzerland and seasonal influenza in general. Namely, we were able to reproduce the following characteristics:

1.) The shape of the epidemic curve was similar in model and extrapolated Swiss sentinel data; overall infection rates and reproduction numbers were in the same range as reported for several outbreaks in various countries.

2.) As we had no empirical data on the age-dependent infection rates for the 2003/2004 H3N2 epidemic in Switzerland, simulated patterns had to be compared with observed patterns from other contexts. Nonetheless, the simulated infection rates and the patterns observed during seven outbreaks in two US municipalities are in good qualitative accordance.

3.) The empirical data show a clear spread along the main transport axes: The epidemic started at the westernmost tip of Switzerland and moved along the main axes to the north-east and from there to the rather isolated canton of Ticino in the south-east. This pattern could be reproduced with our model and median time lags between different cities were in good accordance with the observed data.

With this study, we showed that finding evidence for the validity of influenza models by challenging them with seasonal influenza outbreak data is possible. Validating the structure of influenza models with seasonal influenza data seems us to be a good strategy for making - inherently hypothetical - pandemic models more credible: If a model succeeded in reproducing several aspects of seasonal influenza, it is also likely to generate adequate results for pandemic scenarios.

Further case examples should be reconstructed for gaining more certainty regarding the model's validity. The highest uncertainties in our model lie in the patterns of pre-existing immunity, the proportion and infectiousness of asymptomatic cases and age-dependent attack rates. All these uncertainties could be approached with a longitudinal serological study measuring antibody titres before and after the seasonal influenza period designed as complement to the existing sentinel systems. For better pandemic planning and better informed decisions in the field of seasonal influenza, public health authorities should consider investigating this *terra incognita *in more depth.

## List of abbreviations

CI: 95 percent confidence interval; ILI: influenza-like illness; IQR: inter-quartile range; SD: standard deviation

## Competing interests

The authors declare that they have no competing interests.

## Authors' contributions

TS designed the influenza model, carried out the epidemiological parts of the simulation work, contributed to the quality control of the Swiss MATSim scenario, reviewed the literature and wrote this paper as lead author. MB is one of the head developers of the MATSim project. He carried out the transportation part of the simulation work and helped to draft the manuscript. JH parameterized the infectiousness over time and contributed to the geographical analysis of the simulated data and helped to draft the manuscript. KWA and RWS contributed to the contact network parts of the study design whereas JZ contributed to the disease biology part of the study design. All of them contributed to the manuscript. All authors read and approved the final manuscript.

## Pre-publication history

The pre-publication history for this paper can be accessed here:

http://www.biomedcentral.com/1471-2334/11/115/prepub

## Supplementary Material

Additional file 1 **Additional information on the model parameters and structure**. This supplementary material file provides additional information on several parameters of the simulation and flowcharts describing the central algorithms of the simulation model. Click here for file

Additional file 2 **Sample movie of simulated influenza spread 1**. This movie shows one exemplary simulation run of a simulated seasonal influenza epidemic in Switzerland. The colour scheme illustrates the number of new influenza cases per square kilometre and time step. Click here for file

Additional file 3 **Sample movie of simulated influenza spread 2**. This movie shows one exemplary simulation run of a simulated seasonal influenza epidemic in Switzerland. The colour scheme illustrates the number of new influenza cases per square kilometre and time step. Click here for file

Additional file 4 **Sample movie of simulated influenza spread 3**. This movie shows one exemplary simulation run of a simulated seasonal influenza epidemic in Switzerland. The colour scheme illustrates the number of new influenza cases per square kilometre and time step. Click here for file

Additional file 5 **Sample movie of simulated influenza spread 4**. This movie shows one exemplary simulation run of a simulated seasonal influenza epidemic in Switzerland. The colour scheme illustrates the number of new influenza cases per square kilometre and time step. Click here for file

Additional file 6 **Sample movie of simulated influenza spread 5**. This movie shows one exemplary simulation run of a simulated seasonal influenza epidemic in Switzerland. The colour scheme illustrates the number of new influenza cases per square kilometre and time step. Click here for file

Additional file 7 **Sample movie of simulated influenza spread 6**. This movie shows one exemplary simulation run of a simulated seasonal influenza epidemic in Switzerland. The colour scheme illustrates the number of new influenza cases per square kilometre and time step. Click here for file
