# Supplementary material for: Reconstructing the 2003/2004 H3N2 influenza epidemic in Switzerland with a spatially explicit, individual-based model
Source: BMC Infect Dis. 2011 May 9;11:115. doi: 10.1186/1471-2334-11-115 (PMC3112096; doi:10.1186/1471-2334-11-115)
Supplement: Additional file 1 — Additional information on the model parameters and structure. This supplementary material file provides additional information on several parameters of the simulation and flowcharts describing the central algorithms of the simulation model. [file 1471-2334-11-115-S1.PDF]

Online supplementary material

This supplementary material provides additional information on several parameters of the simulation and the flowcharts describing the central algorithms of the simulation model.

Table of contents

- Parameters
Infectiousness over time....1
Vaccination probability....2
Number of contacts....2
Class size....3
Work group size....3

- Flowcharts
Description of the flowchart symbols....4
Simulation core....5
Infectious seed....6
Generate contact stubs....7
Infect others....8
Stochastic transmission....10
Potential contacts within group....11
Potential contacts (total)....11
Potential contacts neighbor cells....12
Pick contact (total) / within group....13

Infectiousness over time

The following bar chart shows the relative infectiousness over time. The parameters for the corresponding probability function are given in the main text ("Incubation period and infectiousness over time" section). The reference category is the infectiousness of the first day.

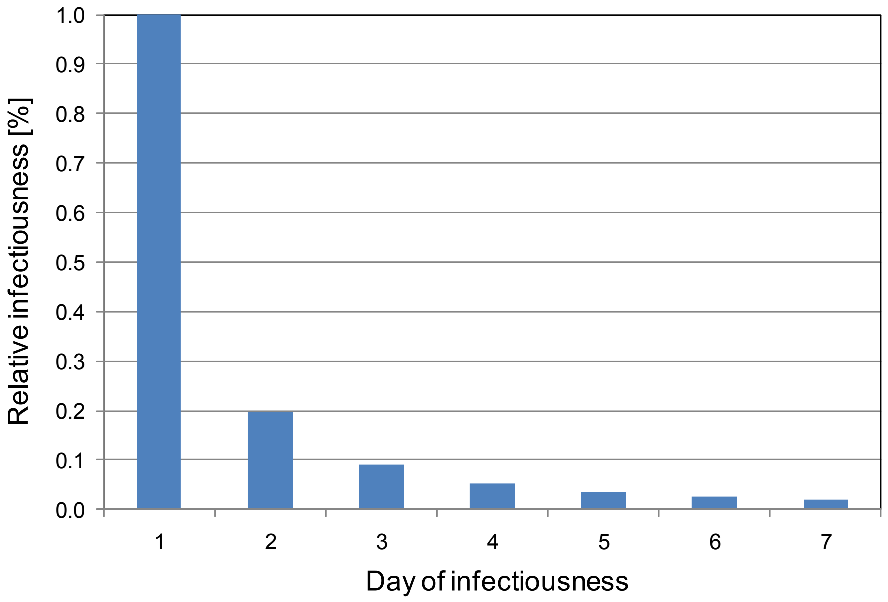

Vaccination probability

The subsequent figure shows the assumed probability of a member of a certain age group to be vaccinated (see “Pre-existing immunity, acquired immunity and vaccination” section in the main text).

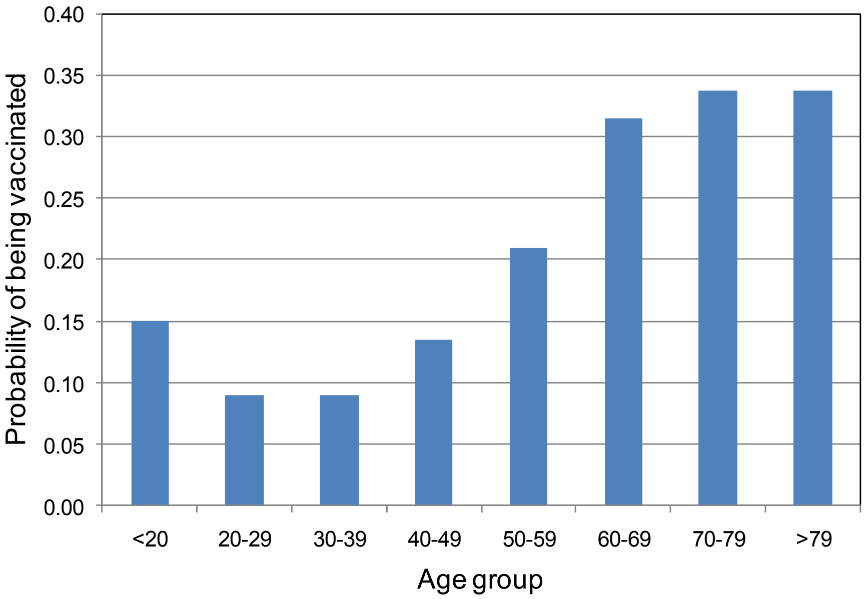

Number of contacts

The following figure shows the degree distribution (distribution of the number of contacts per day) for ten different age groups. These distributions resulted from the household composition by the Volkszählung 2000 (cf. Table 2 of the main text), from the distributions described in Table 4 of the main text, and from the arrangement of activities coming from the MATSim simulation output.

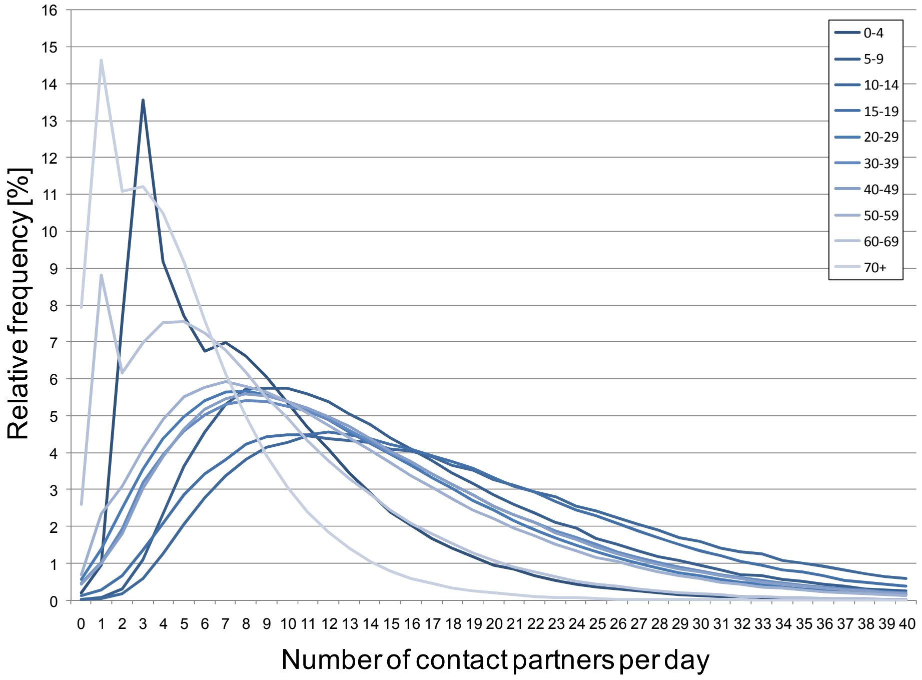

Class size

The following bar chart illustrates the number of students that visit a class of the respective size. The standard class size is 20 students (see also Table 3 of the main text). If there are not enough students of the respective age within one grid cell to fill a class of twenty, smaller classes are created.

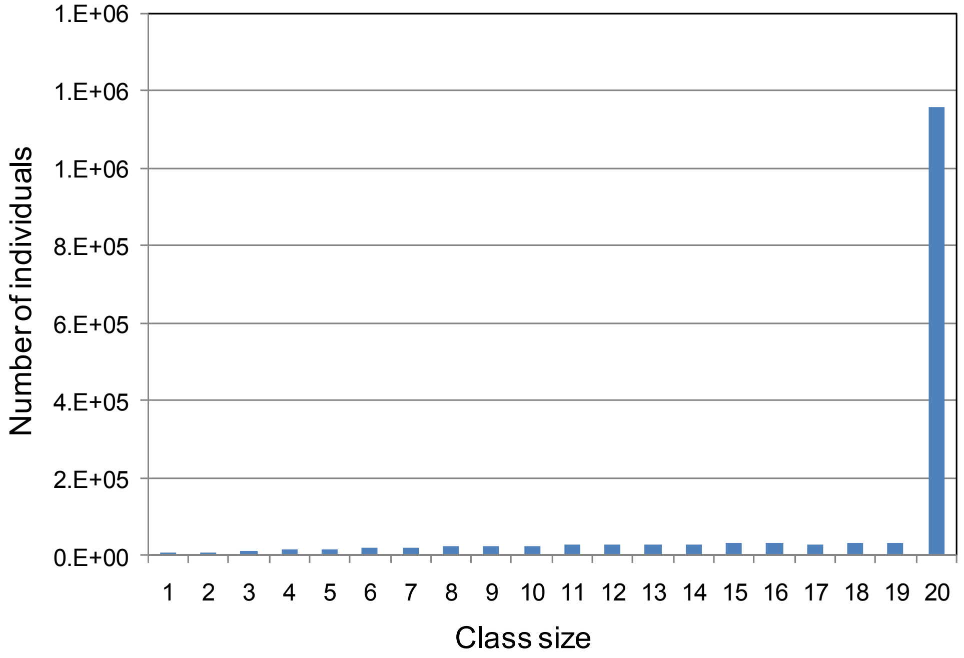

Work group size

The bar chart illustrates the number of individuals that belong to a work group of a certain size (see also Table 3 of the main text).

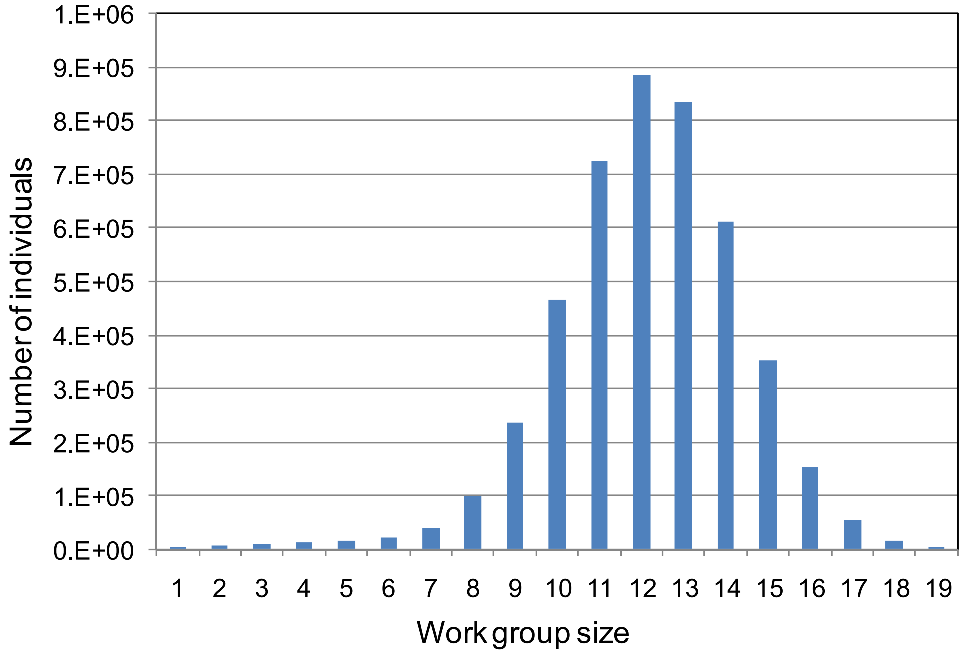

Description of the flowchart symbols

The flowchart symbols used to describe the essential routines of our simulation model are defined in the international standard ISO 5807-1985.

Data

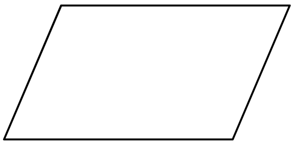

This symbol represents data; the medium is not specified.

Stored Data

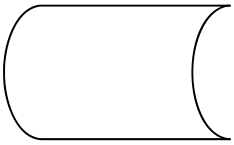

This symbol represents stored data; the medium is also unspecified.

Process

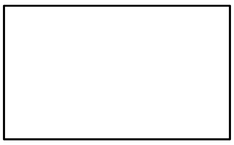

This symbol stands for any kind of process to be executed.

Predefined process

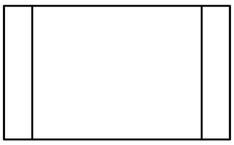

This symbols refers to a process specified elsewhere in the supplementary material.

Loop

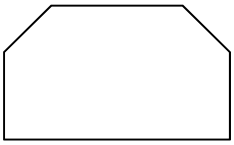

This is the start symbol of a loop.

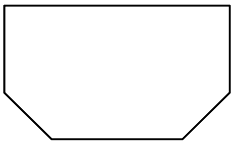

This is the end symbol of a loop. Symbols between the start and the end symbol are executed repeatedly.

Decision

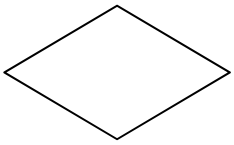

This symbol represents a decision, i.e. the different paths to be chosen, depending on a precisely specified condition.

Simulation core

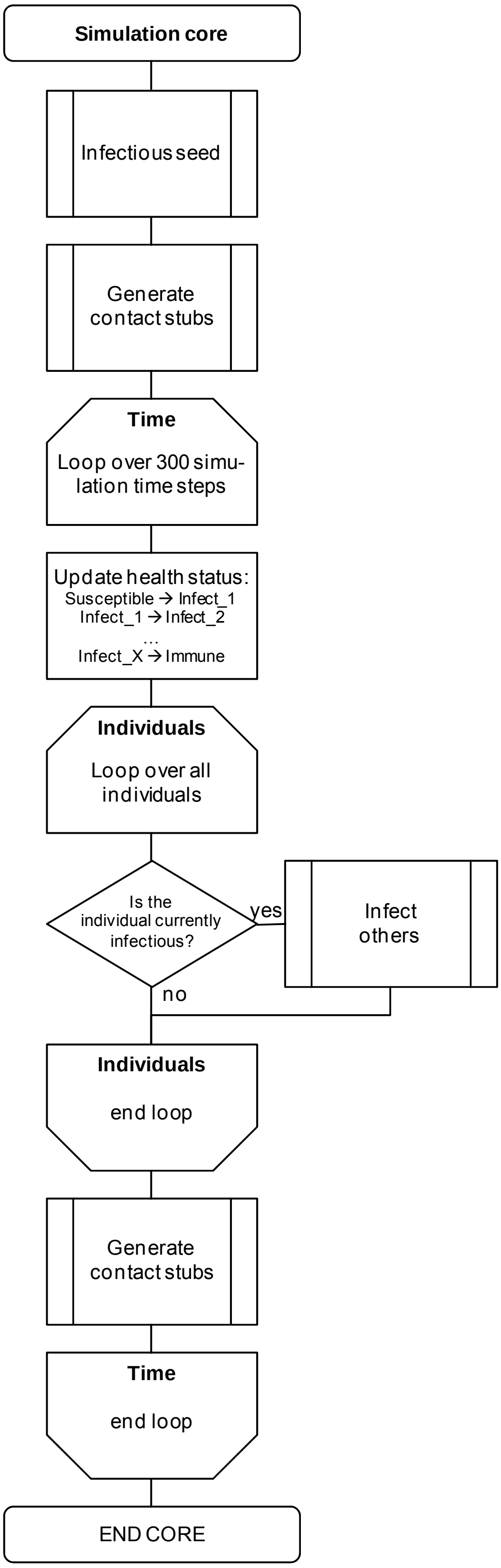

This flowchart shows the core routine of our simulation program. It is a simplified scheme: all preparatory subroutines and all procedures that are not essential for the outcome of the simulation (e.g., I/O-operations, statistics tools, and etcetera) are omitted.

First, the subroutine that generates the infectious seed is called. Then, the stubs needed for linking the population are generated (see "Realizing contacts" section in the main text) for the first simulated day. Every simulation runs 300 iterations, that is, simulated days.

The first procedure done in each iteration step is to update the health status of the entire population: individuals that were infected during the last time step are set to the first day of the status "infectious" (either symptomatic or asymptomatic). All infectious individuals proceed in their individual course of infection. After seven days, all individuals are set to the "immune" state.

Then, all individuals are checked whether they are currently infectious or not. For all infectious individuals, the subroutine "Infect others" is called. Finally, new stubs are generated for the next time step when the "Infect others" routine has been executed for all infectious individuals.

Infectious seed

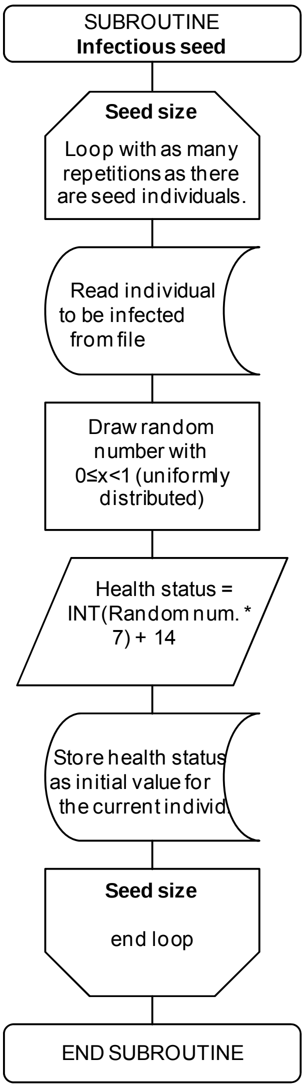

Several different routines for determining the infectious seed are implemented in our program. This flowchart shows the routine used and described in the main text (see "Initial seed" section in the main text).

Agents of the synthetic population that match with reported cases of the sentinel dataset were identified previously and are read from a file. The initial health statuses of these initial cases are determined randomly and can take any value between 20 (1st day of symptomatic infection) and 14 (last day of symptomatic infection).

Generate contact stubs

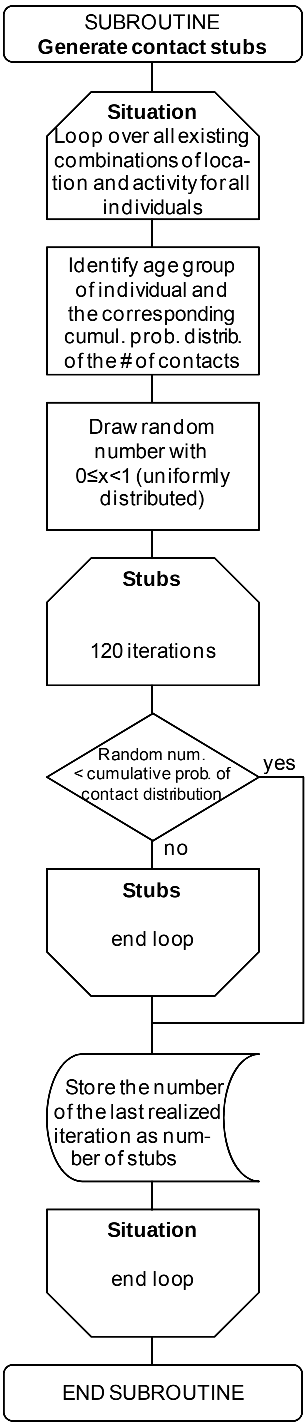

This subroutine generates for every combination of an activity and a location of every individual stubs following the probability distribution described in Table 4 in the main text.

The probability distribution depends on the age group of the respective agent.

Infect others

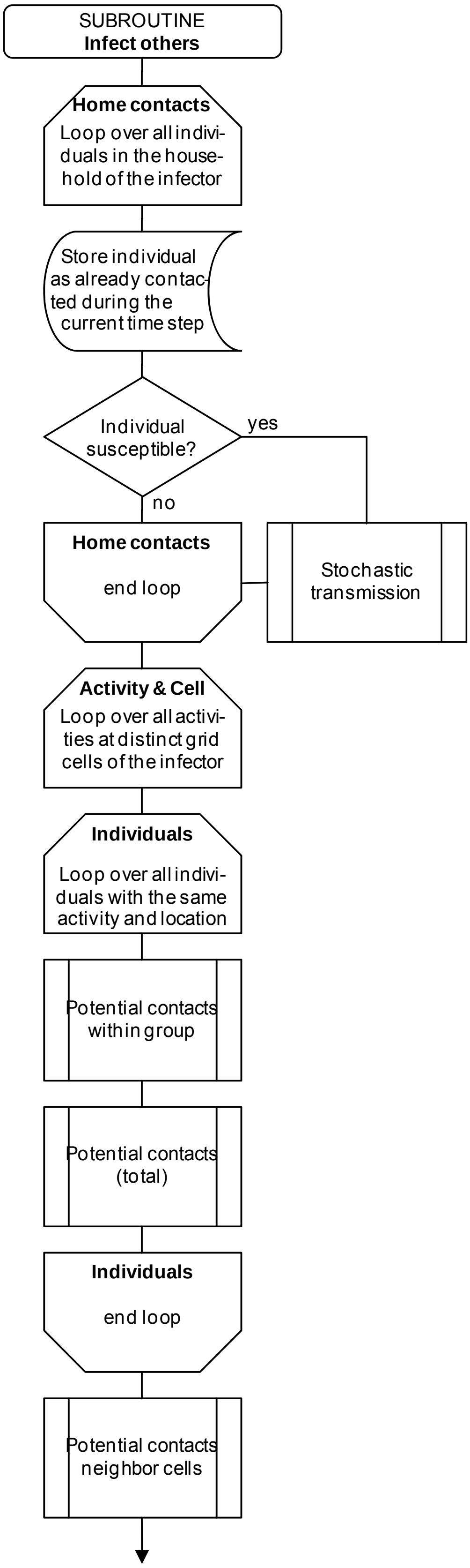

This subroutine determines which other agents are infected by a specific infectious individual. The ID of the current infector is a necessary input for this routine.

First, there is a loop over all individuals who live in the same household as the infector. If such an agent is susceptible, then it is determined stochastically whether the contact partner will be infected or not. All members of the infector's household are flagged as already contacted during the current time step in order to prevent double contacting.

Then, there is a loop over all activities of the infector that take place at distinct locations. For every combination of activity and grid cell, all other individuals who perform the same activity within the same grid cell are identified. With a loop over all these individuals, it is identified whether an individual belongs to the same pre-defined group as the infector (e.g., classes at school or work groups) or not. Individuals who share an activity and location with the infector but do not have the same group membership as the latter are stored in a list of all potential contact partners. Additionally, individuals who are members of the group in which the infector belongs are stored in a list of potential group contact partners.

If not enough potential contact partners can be identified who share an activity **and** a grid cell with the infector, then the individuals of neighboring grid cells are also considered as potential contact partners.

After the identification of the infector's potential contact partners for a certain combination of grid cell and activity, all unconnected contact stubs of the infector become consecutively connected within the loop "target contacts". For every stub, a random number is first drawn. Then, with a probability of .75, the contact partner is chosen from the set of potential partners within the group (if there are elements left in this set); and, with a probability of .25, a contact partner is chosen from the set of all potential contact partners.

If the chosen contact partner is susceptible, then it is determined stochastically whether the contact partner will be infected or not. In the next step, the chosen individual

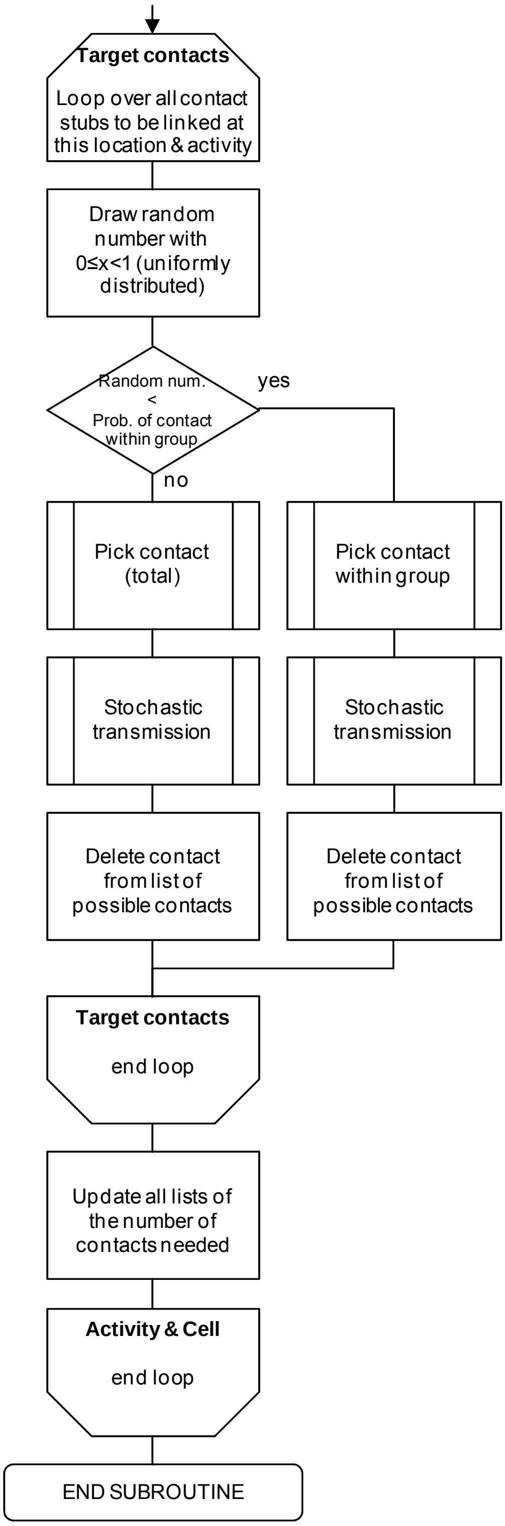

is deleted from the list of eligible contact partners to ensure that this specific partner cannot be chosen again by the same infector within the same time step.

Finally, the number of unconnected stubs for all involved agents – the infector and the chosen contact partners – is reduced by the respective number of connections made.

Stochastic transmission

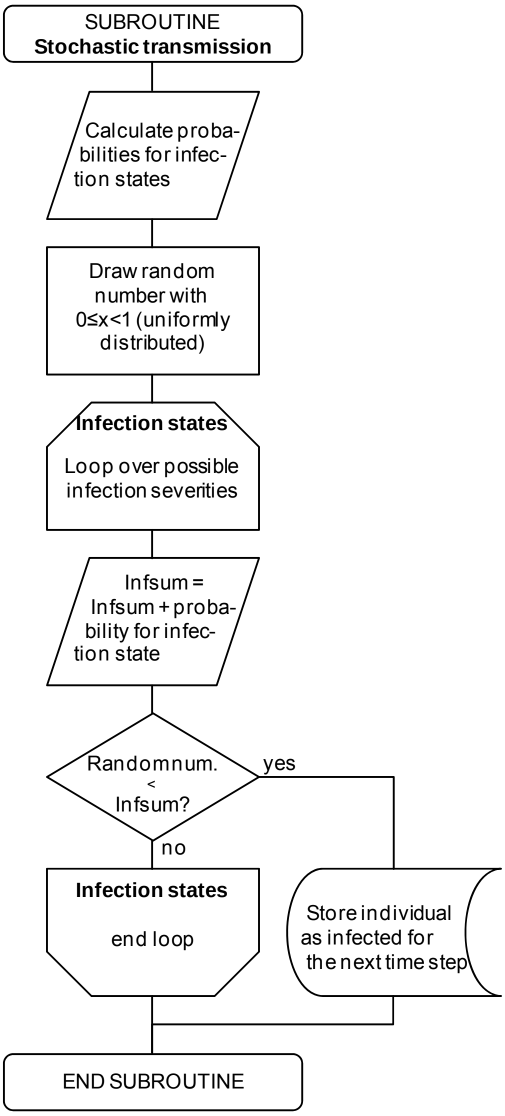

The subroutine "Stochastic transmission" determines stochastically for susceptible contact partners of an infector, whether these contact partners become infected by this specific infector or not.

First, the probabilities for the two outcomes, (i) symptomatic and (ii) asymptomatic infection, are calculated. Then, a random number is drawn. In a loop over all possible infection severity states (here just (i) and (ii) as described above), it is determined whether the contact partner of the infector will be symptomatically or asymptomatically infected in the next time step or whether the contact partner will remain susceptible.

Potential contacts within group

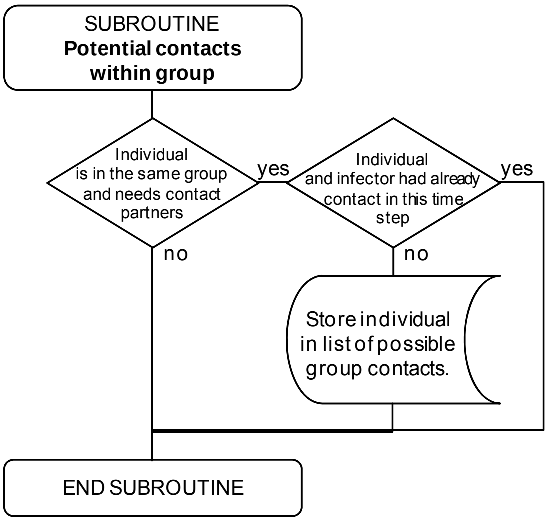

This subroutine identifies agents who perform the same activity as the infector within the same grid cell and who belong to the same group (i.e., school class or work group).

It is checked whether the current agent under consideration belongs to the same group as the infector, whether it has unconnected contact stubs for the current activity and location, and whether it had no previous contact with the infector in the current time step. If all of these conditions are fulfilled, the agent is stored in a list of potential group contact partners.

Potential contacts (total)

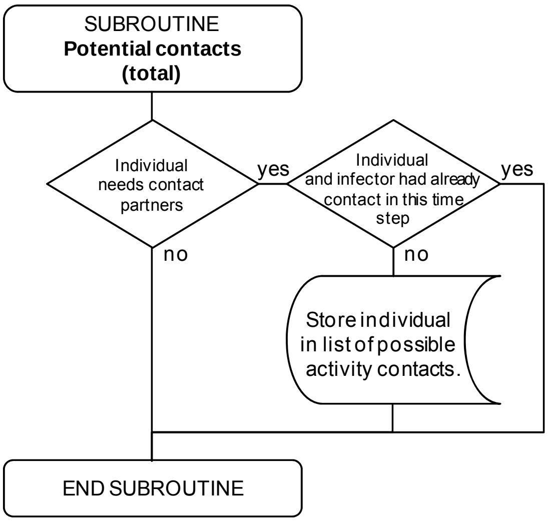

Analogous to “Potential contacts within group”.

Potential contacts neighbor cells

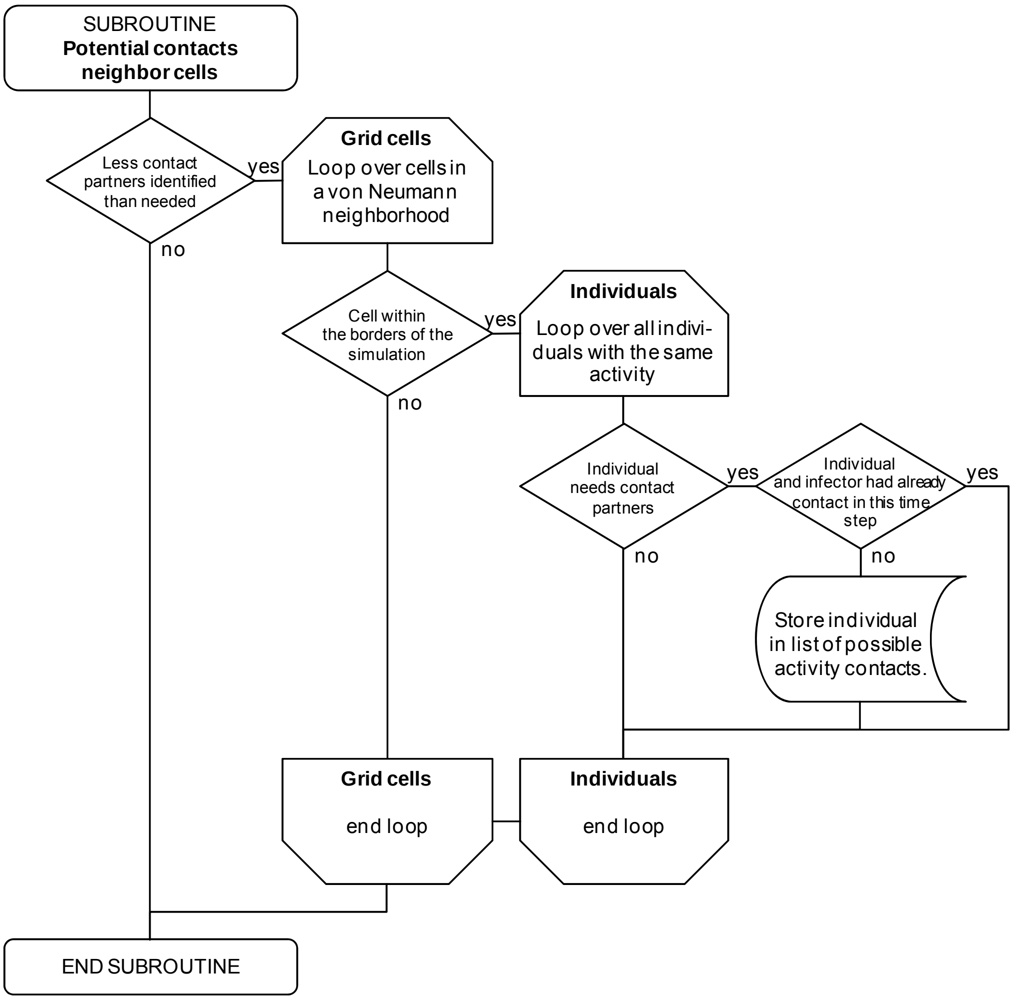

If not enough contact partners can be identified to connect all unconnected contact stubs of the infector, individuals performing the same activity in surrounding cells are also considered.

The subroutine performs a loop over all surrounding cells (von Neumann neighborhood) and over all individuals within these cells. If such an individual still has unconnected contact stubs for the respective activity and if it had no previous contact with the infector during the current time step, it is stored in the list of potential contact partners.

Pick contact (total) / within group

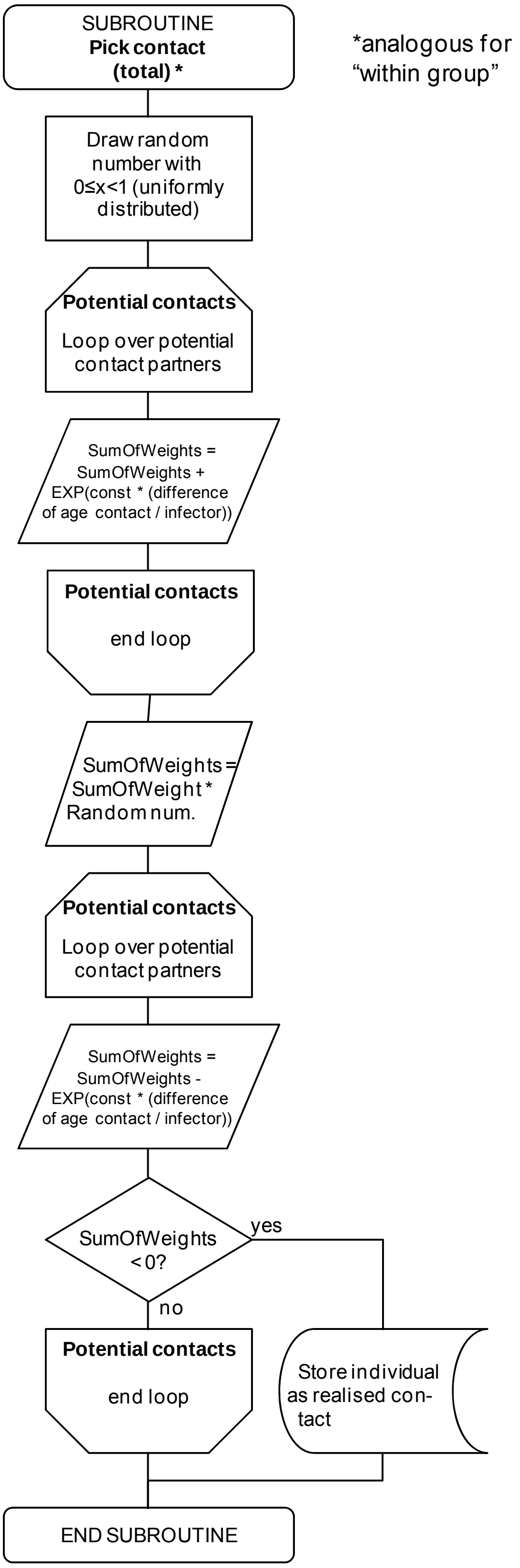

The two subroutines "Pick contact (total)" and "Pick contact within group" chose agents from the lists of potential contact partners as actually realized contacts.

In a loop over all potential contact partners, both individual probability weights and the sum of these weights are calculated. These weights depend on the age difference between infector and potential contact as described in the main text.

The sum of weights is multiplied with a random number between 0 and 1. In a second loop over all potential contact partners, the individual probability weights are subtracted from the reduced sum of weights. The individual for which the sum of weights drops below zero is chosen as a realized contact.
